# The impact of academic burnout on academic achievement: a moderated chain mediation effect from the Stimulus-Organism-Response perspective

**DOI:** 10.3389/fpsyg.2025.1559330

**Published:** 2025-06-05

**Authors:** Zhenkai Qin, Guifang Yang, Ziqian Lin, Yimin Ning, Xiaolong Chen, Hongfeng Zhang, Cora Un In Wong

**Affiliations:** ^1^School of Information Technology, Guangxi Police College, Nanning, China; ^2^School of Business Administration, Guangxi University, Nanning, Guangxi, China; ^3^School of Public Administration, Guangxi Police College, Nanning, China; ^4^School of Mathematical Sciences, East China Normal University, Shanghai, China; ^5^Faculty of Humanities and Social Sciences, Macao Polytechnic University, Macau, China

**Keywords:** academic burnout, academic achievement, learning satisfaction, learning engagement, educational values

## Abstract

**Objective:**

With the increasing academic pressure faced by university students, academic burnout has gradually become a critical factor affecting students' learning outcomes, drawing widespread attention in the field of education.

**Methods:**

A survey was conducted among 306 Chinese university students using five validated instruments: the Academic Burnout Scale, Learning Satisfaction Scale, Learning Engagement Scale, Academic Achievement Scale, and Educational Values Scale. Data were analyzed using SPSS 26.0 and PROCESS macro (Model 6 and Model 14), incorporating correlation analysis, regression analysis, mediation and moderated mediation tests via bootstrapping.

**Results:**

Academic burnout had a significant negative effect on academic achievement. Both learning satisfaction and learning engagement had significant positive effects on academic achievement and jointly played a chain mediation role between academic burnout and academic achievement. Educational values significantly moderated the effect of learning engagement on academic achievement, as well as the entire chain mediation pathway.

**Discussion:**

The results highlight a significant chain mediation effect of learning satisfaction and engagement in the relationship between academic burnout and academic achievement. Moreover, educational values can significantly moderate the relationship between academic burnout and learning achievement. Based on the empirical findings, the study proposes three recommendations for improving students' learning achievements: Improve students' learning satisfaction; Strengthen the development of students' learning engagement; Enhance the positive influence of educational values. By addressing academic burnout from the perspectives of learning satisfaction, learning engagement, and educational values, the study aims to improve students' learning achievements and foster collaboration between schools, teachers, and students to create positive conditions for academic development.

## 1 Introduction

In today's highly competitive academic landscape, where the pressures of both internal expectations and external demands continually grow, university students often find themselves trapped in the debilitating cycle of academic burnout. This phenomenon has become an urgent concern, as it stands as a major impediment to effective learning and academic success (Barbayannis et al., 2022). Reducing academic burnout has thus become a pressing challenge in contemporary educational discourse. The concept of academic burnout was first introduced in 1992 by W. Bruce Schaufeli, offering a foundational framework for understanding how students, much like professionals, can suffer from burnout within the academic setting (Carmona-Halty et al., 2024).

Freudenberger and Maslach's definition of burnout is widely recognized, describing it as a prolonged state of stress and negativity toward academic tasks, which drains a student's energy and undermines their motivation (Hillert et al., 2020). Jan further emphasized that academic burnout stems from sustained academic pressures, leading students to feel detached from their studies, manifesting in a reluctance to invest time and energy. This, in turn, cultivates feelings of meaninglessness, diminished self-worth, and a noticeable drop in academic performance (Tao et al., 2024). For this research, we adopt Jan's perspective on burnout, providing a lens through which we analyze its dynamics.

Recent longitudinal studies have emphasized that academic burnout is not static but develops and fluctuates over time, with sustained impacts on students' motivation, learning engagement, and academic performance (Zhan and Yksel, 2021); see also Parviainen et al. (2021) and Hong et al. (2025). These studies provide empirical support for examining the time-dependent mechanisms of burnout and its downstream effects on learning.

The academic exploration of burnout has been rapidly evolving, with a focus on both its underlying causes and its consequences on learning outcomes (Puah et al., 2024). Existing studies can be categorized into three broad themes: the development and validation of measurement tools for academic burnout (Messina et al., 2024); investigations into contributing factors such as learning goals, study methods, and personality traits (Deng et al., 2022); and the impact of burnout on outcomes such as learning satisfaction, engagement, and self-efficacy (Yang et al., 2022). Additionally, cross-cultural comparisons have shown that educational systems and cultural contexts can shape how academic burnout is experienced and coped with, especially in collectivist versus individualist societies (Cabras et al., 2023); see also Tao et al. (2024). These diverse studies collectively form a comprehensive understanding of academic burnout and provide essential insights for educational policy.

Despite the growing attention to academic burnout, few studies have systematically examined how multiple psychological mechanisms jointly influence its impact on academic achievement. To address this gap, the present study introduces the Stimulus-Organism-Response (SOR) framework, which allows the integration of both chain mediation and moderation effects into a unified model. Specifically, we investigate the mediating roles of learning satisfaction and learning engagement, as well as the moderating role of educational values–defined as students' beliefs regarding the importance, meaning, and utility of education (Yu et al., 2024). Although academic burnout has become increasingly prominent among students in Asian universities–particularly in China–few studies in this context have applied such integrated models. By grounding this research in the Chinese higher education system, we aim to extend previous literature and offer culturally relevant insights into the mechanisms linking burnout and achievement. This approach not only deepens the theoretical understanding of academic burnout but also provides evidence-based recommendations for improving student outcomes in culturally specific educational environments.

## 2 Theoretical foundation and research hypotheses

### 2.1 Variable definitions

**Academic burnout** is defined as an emotional and cognitive exhaustion state resulting from prolonged academic pressure and workload, often manifesting as emotional fatigue, disengagement from academic activities, and negative cognitive appraisals of learning tasks (Kourea et al., 2023).

**Learning satisfaction** is a positive psychological state that arises from students' perceptions and evaluations of their educational experience and outcomes. It reflects the extent to which students' desires and needs are met throughout their academic journey and represents their overall attitude toward the learning process (Olson et al., 2023). This satisfaction encompasses cognitive and emotional responses to various elements of the learning experience, including the course content, teaching methods, teacher-student interaction, and the learning environment (Nguyen et al., 2024). In this model, learning satisfaction is placed before learning engagement, as emotional responses to academic experiences typically precede behavioral reactions such as engagement. This ordering aligns with existing motivational theories.

**Learning engagement** is a persistent, immersive, and positive behavioral state related to learning or academic research, characterized by vigor, dedication, and concentration (Ma and Wei, 2022). Students who are engaged in their studies typically show high levels of motivation and commitment, while disengaged students demonstrate apathy, indifference, or even hostility toward academic tasks (Zhao et al., 2021).

### 2.2 Relationships between variables

#### 2.2.1 The relationship between academic burnout and academic achievement

Under academic burnout, students are likely to experience emotional depletion, loss of motivation, and a decline in their academic self-efficacy, resulting in the inability to achieve the expected academic outcomes despite continued academic effort (Xu et al., 2022). These negative psychological symptoms cause students to reduce their academic aspirations, weakening their learning interest and motivation.Burnout not only reduces motivation but also leads to emotional avoidance behaviors, diminishing participation in academic tasks (Qiang et al., 2024). As a result, academic burnout directly leads to reduced learning engagement, significantly lowering academic performance and overall achievement (Li et al., 2024).

#### 2.2.2 The mediating role of learning satisfaction

Burnout reduces this satisfaction by weakening students' emotional state and lowering their perceived value of learning, often resulting in a loss of interest and motivation (Zhang et al., 2024). The sequence posits that emotional dissatisfaction, as part of learning satisfaction, occurs prior to behavioral disengagement or reduced learning engagement, following well-established causal pathways in motivational theory. Students with higher levels of satisfaction tend to demonstrate stronger engagement and better academic outcomes (Liu et al., 2024).

#### 2.2.3 The mediating role of learning engagement

Academic burnout significantly undermines students' engagement, both emotionally and behaviorally, thereby reducing learning effectiveness and performance (Li, 2022). The extent of engagement plays a pivotal role in determining academic achievement (Wu et al., 2021).

#### 2.2.4 The chain mediating role of learning satisfaction and learning engagement

Academic burnout is often manifested as irritability, frustration, and emotional exhaustion. In academic settings, it can lead to physical and mental fatigue, affecting students' learning processes (Xu and Ba, 2022). Academic burnout (a negative stimulus) can trigger emotional fatigue (learning satisfaction) and cognitive overload (learning engagement). When students experience academic burnout, it initiates emotional exhaustion, leading to negative feelings about academic tasks, thereby lowering learning satisfaction. This reduction in satisfaction further reduces students' learning motivation and engagement, ultimately impairing academic performance and achievement. This causal sequence–where emotional dissatisfaction precedes behavioral disengagement–is also supported by prior research (Nguyen et al., 2024).

Thus, academic burnout exerts its influence through a chain mediation effect: first affecting learning satisfaction, which in turn impacts learning engagement, eventually leading to lower academic performance (Zhang et al., 2021).

#### 2.2.5 The moderating role of educational values

Studies have shown a negative correlation between learning burnout and educational values. Research by Ge Yu found that educational values can negatively predict learning burnout. Students who hold vague or negative values tend to experience higher academic stress, leading to greater burnout (Yu et al., 2024). This finding aligns with the work of Santos, who noted that positive values can effectively reduce the negative impact of academic stress on students and enhance their coping abilities, thus reducing the likelihood of burnout (de la Fuente et al., 2021). Furthermore, Zhang's research confirmed the positive effect of educational values in boosting students' engagement and academic success. Students with clear and positive educational values tend to exhibit higher levels of learning engagement, which significantly enhances their academic achievements (Jian, 2022).

### 2.3 Development of hypotheses

**H1:** Academic burnout has a significant negative impact on academic achievement.

**H2:** Learning satisfaction mediates the relationship between academic burnout and academic achievement.

**H3:** Learning engagement mediates the relationship between academic burnout and academic achievement.

**H4:** Learning satisfaction and learning engagement play a chain mediating role in the relationship between academic burnout and academic achievement.

**H5:** Educational values moderate the impact of learning engagement on academic achievement.

**H6:** Educational values moderate the chain mediating effect of learning satisfaction and learning engagement on academic achievement.

### 2.4 Stimulus-Organism-Response (SOR) theory

The Stimulus-Organism-Response (SOR) framework, introduced by Mehrabian and colleagues, posits three critical components: stimulus, organism, and response (Nagano et al., 2023). In this model, the *stimulus* serves as the independent variable, representing the external environmental factors that influence an individual's psychological state. The *organism*, acting as a mediator, refers to internal psychological processes, including emotional reactions and cognitive appraisals. Finally, the *response* is the dependent variable, manifesting as the impact of environmental factors on individual behaviors, typically in the form of approach or avoidance (Gao et al., 2022).

According to SOR theory, academic burnout (stimulus, S) affects students' academic achievement (response, R) through learning satisfaction (O, emotional-cognitive) and learning engagement (O or R, behavioral-cognitive) (Bekker et al., 2023). Specifically, academic burnout triggers emotional fatigue and psychological exhaustion, diminishing students' positive emotional responses and satisfaction with their studies. This decline in learning satisfaction further reduces students' motivation and engagement in learning, leading to decreased academic performance. Learning engagement, acting as a bridge between emotional cognition and behavioral outcomes, weakens as burnout increases, directly impairing students' academic performance and learning achievements. This chain mechanism illustrates how academic burnout negatively impacts academic success through emotional and behavioral pathways, providing a theoretical basis for interventions aimed at alleviating its detrimental effects, such as enhancing learning satisfaction and engagement.

This study draws upon the SOR theory to explore the mediation and moderation effects of various factors influencing the relationship between academic burnout and academic achievement, shedding light on the underlying mechanisms at play.

### 2.5 Conclusion

**Figure 1:**
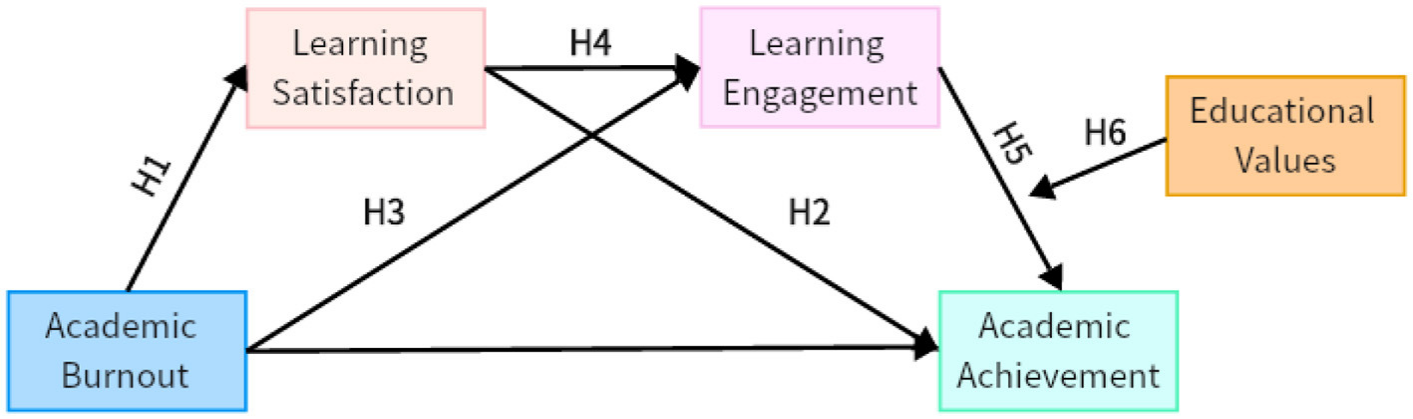
Chain mediation model diagram.

In summary, learning satisfaction, learning engagement, and academic achievement not only directly influence academic burnout but are also interrelated. It is hypothesized that a chain mediation effect exists in the relationship between academic burnout, learning satisfaction, learning engagement, and academic achievement, which is briefly illustrated in Figure 1 to enhance clarity.

## 3 Study subjects and methods

### 3.1 Research model

Study constructs a moderated chain mediation model based on the Stimulus-Organism-Response (SOR) theory. As shown in Figure 1, the model posits that academic burnout affects academic achievement through the mediating roles of learning satisfaction and learning engagement. Meanwhile, educational values moderate the effect of learning engagement on achievement, as well as the overall chain mediation pathway.

### 3.2 Study subjects and sample selection

#### 3.2.1 Sample population

This study employed a survey-based approach, with the study subjects being undergraduate students. Participants were required to be full-time undergraduate students currently enrolled in a standard four-year academic system, having completed at least three months of university study. Based on the guideline of having at least 5 participants per predictor variable, this study includes five key predictors. To ensure sufficient statistical power for regression analysis and mediation testing, a total of 306 university students were selected as the research subjects.

#### 3.2.2 Selection criteria

The selection criteria for participants were as follows: participants must be undergraduate students currently enrolled in a full-time university program; they must be in a standard four-year academic system; they must have been enrolled for at least three months. After excluding incomplete responses and those with identical answers across all items, 306 valid questionnaires were retained, yielding an impressive response rate of 98.7%.

### 3.3 Research tools

The study's variables and measurement indicators were derived from a demographic information sheet and five established scales, with a total of 46 items. The five scales were chosen because they align with the core variables of the SOR model and have demonstrated good reliability in previous research. The academic burnout scale has been widely used in Schaufeli et al. (2002), and its high internal consistency in the Chinese educational context was also validated in Zheng et al. (2024). These scales have been validated or adapted within the Chinese educational context. To ensure cultural relevance, we referred to Teuber et al. (2021), who emphasized the need for appropriate modifications when using scales in the Chinese educational environment to improve clarity. Based on a sample of Chinese university students, we made minor adjustments to the wording of the scale items. A pilot study with 50 participants was conducted prior to data collection, showing good internal consistency (Cronbach's α = 0.850). These participants were excluded from the final sample, confirming the questionnaire's suitability for the main study.

#### 3.3.1 Control variables design

A demographic information sheet was designed as a control variable, collecting data on characteristics such as gender, academic year, and place of origin. By controlling for these factors, the study ensures that the observed effect of academic burnout on academic achievement is not confounded by other variables, thus improving the accuracy and interpretability of the results.

#### 3.3.2 Scale selection

The study used five well-established scales, employing a 5-point Likert scale for all items. The Likert scale data were treated as continuous variables in the analysis. The details of the scales are as follows:

**Academic burnout scale**: This scale, based on Lian Rong's student burnout questionnaire (Carmona-Halty et al., 2024). Nine items were adapted for use in this study, such as “I feel exhausted when I think about facing a day of study.” Participants rated each item on a 5-point scale, where 1 = “Strongly disagree” and 5 = “Strongly agree.” Higher scores indicate greater academic burnout. The Cronbach's α for this scale was 0.911 in this study.

**Learning satisfaction scale**: Based on Xu Qianhui's student learning satisfaction scale (Topala and Tomozii, 2014). Nine items were adapted for use in this study, such as “Satisfaction with library resources and services.” Respondents rated each item using a 5-point scale, where 1 = “Very dissatisfied” and 5 = “Very satisfied.” Higher scores indicate greater learning satisfaction. The Cronbach's α for this scale was 0.925.

**Learning engagement scale**: This scale, developed by Fang Laitan, Shi Kan, and others (Dauzón-Ledesma and Izquierdo, 2023), included nine items such as “I look forward to studying as soon as I wake up.” The 5-point Likert scale was used, with higher scores indicating higher engagement in learning. The Cronbach's α for this scale was 0.925.

**Academic achievement scale**: Based on Wang Qiuhua's 2001 learning achievement scale (Luo et al., 2023). Six items were adapted for use in this study like “I am excited to study the moment I wake up.” Some items were reverse-scored. Higher scores on this scale indicate better academic achievement. The Cronbach's α for this scale was 0.872.

**Educational value scale**: This scale was adapted from the educational value scale developed by Niu Chunjuan and Zheng Yong (Seetee et al., 2020). Nine items were adapted for use in this study, such as “It's better to work or start a business than attend university”. Some items were reverse-scored. Higher scores indicate stronger educational values. The Cronbach's α for this scale was 0.907.

All five scales exhibited Cronbach's α values greater than 0.800, demonstrating exceptional internal consistency and reliability. These scales consistently reflected the research variables, minimizing error and enhancing the precision of data analysis. Therefore, the data collected using these scales provided strong support for the subsequent experimental analysis and hypothesis testing, ensuring the scientific validity of the research conclusions. Additionally, the results confirm that the questionnaire design phase effectively ensured the measurement tools' quality, providing a solid foundation for the study's progress.

### 3.4 Research methodology

The data collected were analyzed using SPSS 26.0 and SPSSAU software. The analysis involved tests for common method bias, correlation analysis, mediation effect testing, and moderation effect analysis. A significance level of *p* < 0.05 was considered statistically significant, confirming the relevance of the observed relationships.

## 4 Results and analysis

### 4.1 Common method bias

In this study, we employed Harman's single-factor test (Pan and Yuan, 2023) to mitigate the potential common method bias introduced by the questionnaire survey. The analysis revealed that eight factors had eigenvalues greater than 1. Of particular note, the first factor explained 32.488% of the total variance, which is well below the 40% threshold commonly accepted as a benchmark. This suggests that common method bias does not significantly affect our results, enabling us to proceed with further analysis. The details of this test are presented in Table 1, which provides the eigenvalues and variance explained by each factor to support the conclusion.

**Table 1:** Common method bias test.

**Components**	**Initial Eigenvalues**			**Extraction sum of squares loadings**		
	**Total**	**Percentage of variance (%)**	**Cumulative (%)**	**Total**	**Percentage of variance (%)**	**Cumulative (%)**
1	17.704	38.488	38.488	17.704	38.488	38.488
2	2.901	6.307	44.795	2.901	6.307	44.795
3	2.110	4.587	49.382	2.110	4.587	49.382
4	1.647	3.581	52.962	1.647	3.581	52.962
5	1.376	2.992	55.954	1.376	2.992	55.954
6	1.193	2.593	58.547	1.193	2.593	58.547
7	1.136	2.469	61.016	1.136	2.469	61.016
8	1.055	2.294	63.310	1.055	2.294	63.310
9	0.965	2.099	65.409	-	-	-

### 4.2 Correlation analysis

Correlation analysis stands as one of the most widely utilized techniques in the realm of relational studies. In essence, it examines whether a linear relationship exists between two variables. The findings from this study's correlation analysis are displayed in Table 2, which provides descriptive and correlational evidence supporting the relationships between core variables. The data reveal a striking pattern: academic burnout in students is significantly negatively correlated with both learning satisfaction and learning engagement, as well as with academic achievement. On the other hand, learning satisfaction and learning engagement both exhibit a positive correlation with academic achievement. Furthermore, the educational values of students are positively associated with their academic performance. However, while these correlations are evident, the underlying causal relationships, pathways, and mechanisms between the variables require a more comprehensive investigation using structural equation modeling for deeper validation and insight.

**Table 2:** Descriptive statistics and correlation analysis.

	**AB**	**LS**	**LE**	**AA**	**EV**
AB	1				
LS	-0.670**	1			
LE	-0.726**	0.661**	1		
AA	-0.632**	0.604**	0.663**	1	
EV	-0.571**	0.539**	0.511**	0.612**	1

**Correlation is significant at the 0.01 level (two-tailed). In this table, **AB** represents Academic Burnout, **LS** represents Learning Satisfaction, **LE** represents Learning Engagement, **AA** represents Academic Achievement, and **EV** represents Educational Values.

### 4.3 Regression analysis

#### 4.3.1 Multicollinearity diagnostics

Before conducting the mediation analysis, we performed multicollinearity diagnostics. As shown in Table 3, the Variance Inflation Factor (VIF) values for all predictors ranged from 1.04 to 2.69, indicating that there is no significant multicollinearity among the variables in the model. This further confirms the statistical validity of the model and ensures the reliability of the regression analysis results.

**Table 3:** Multicollinearity diagnostics using VIF.

**Variable**	**VIF**
Academic burnout (AB)	2.56
Learning satisfaction (LS)	2.15
Learning engagement (LE)	2.69
Educational values (EV)	2.04
Interaction (LE × EV)	1.04

#### 4.3.2 Testing for mediation effects

**Figure 2:**
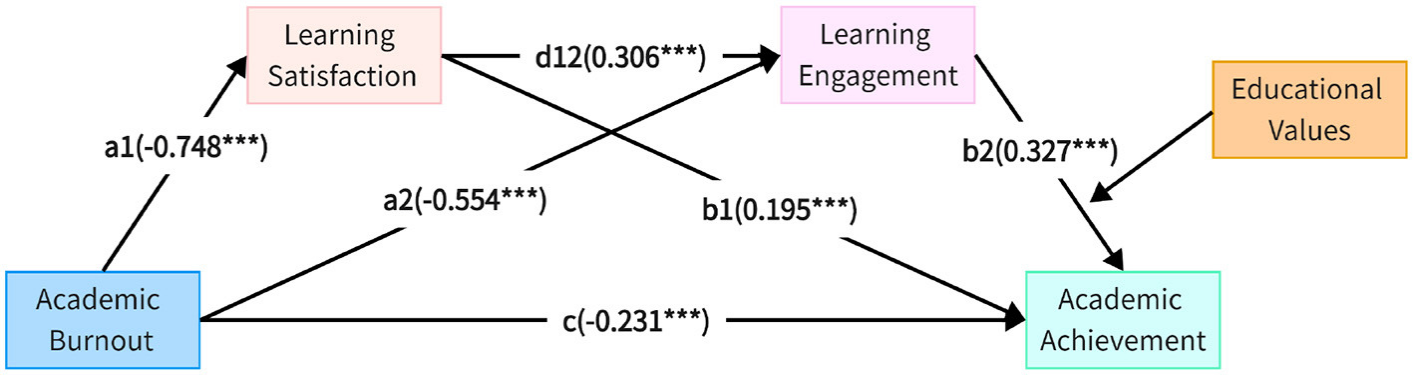
Chain mediation effect model diagram. ^***^ Indicates *p* < 0.001, denoting a highly significant effect.

In this study, we combine path analysis with Bootstrap sampling to rigorously examine the chain mediation effects. Initially, the PROCESS 3.0 plugin is employed to perform path analysis, establishing a chain mediation model and verifying the influence paths within it. These paths are analyzed through standardized regression coefficients to reveal the direct, indirect, and total effects between variables. Furthermore, we leverage Bootstrap sampling (with 5,000 repetitions) to calculate the confidence intervals for the indirect effects, thus ensuring the robustness and reliability of the results. The outcomes are detailed in Figure 2 and Table 4, which visualize and quantify the tested mediation effects.

**Table 4:** Regression analysis of chain mediation effects.

**Predictor variables**	**Learning satisfaction**		**Learning engagement**		**Academic achievement**	
	**Coefficient**	***t*** **-value**	**Coefficient**	***t*** **-value**	**Coefficient**	***t*** **-value**
Constant	5.2800	44.6666	3.8048	13.9042	2.5047	7.0896
Academic burnout	-0.7476**	-15.7303	-0.5544**	-10.2896	-0.2309**	-3.6574
Learning satisfaction			0.306**	6.3382	0.1952**	3.7665
Learning engagement					0.3272**	5.6455
R^2^	0.4487***		0.5829***		0.5103***	
F	247.442		211.709		104.9165	

^**^ Indicates *p* < 0.01, suggesting statistical significance at the 99% confidence level (strong significance); ^***^ indicates *p* < 0.001, suggesting statistical significance at the 99.9% confidence level (very strong significance).

**(1) Path 1: “Academic burnout**→**Learning satisfaction**→**Learning achievement”**

As presented in Table 4, the path coefficient from academic burnout to learning achievement is 0.231, with a *p*-value of 0.0003, indicating a negative relationship where higher burnout leads to lower academic achievement. This indicates a significant, positive relationship between academic burnout and learning achievement. Meanwhile, the path from academic burnout to learning satisfaction is -0.7476, with a *p*-value of 0.0000, pointing to a robust negative influence. Lastly, the path from learning satisfaction to learning achievement shows a coefficient of 0.1952, with a *p*-value of 0.0000, demonstrating a statistically significant positive impact of learning satisfaction on academic success.

Table 5 further reveals that the direct effect of academic burnout on learning achievement is -0.231, with a 95% confidence interval ranging from [-0.355, -0.107] – an interval that excludes zero, confirming a strong negative correlation. This supports Hypothesis H1. In addition, the effect size for Path 1 is -0.146, with a 95% confidence interval from [-0.273, -0.034], which also does not encompass zero, solidifying the significant mediating role of learning satisfaction. In essence, academic burnout weakens students' positive evaluations and their satisfaction with learning, ultimately leading to a drop in learning achievement. Therefore, Hypothesis H2 holds true.

**Table 5:** Chain mediation pathways.

**Pathway type**	**Mediation pathway**	**Effect value**	**Bootstrapping**
Indirect effect	**Pathway 1**: Academic burnout → Learning satisfaction → Academic achievement	-0.146	[-0.273, -0.034]
	**Pathway 2**: Academic burnout → Learning engagement → Academic achievement	-0.181	[-0.300, -0.081]
	**Pathway 3**: Academic burnout → Learning satisfaction → Learning engagement → Academic achievement	-0.075	[-0.135, -0.028]
Direct effect	**Pathway 4**: Academic burnout → Academic achievement	-0.231	[-0.355, -0.107]

**(2) Path 2: “Academic burnout**→**Learning engagement**→**Learning achievement”**

Turning to Table 4, we observe that the path from academic burnout to learning engagement is -0.5544, with a *p*-value of 0.000, underscoring a significant negative effect. Similarly, the path from learning engagement to learning achievement stands at 0.3272, with a *p*-value of 0.0000, reinforcing that greater engagement in learning positively influences academic performance.

Table 5 shows that the effect size for Path 2 is -0.181, with a 95% confidence interval between [-0.300, -0.081], excluding zero, which highlights the significant mediating role of learning engagement. Essentially, academic burnout dampens students' engagement levels, leaving them with insufficient energy and initiative to invest in their learning, which results in lower academic achievement. This indicates that burnout leads to disengagement, which in turn lowers academic achievement. Thus, Hypothesis H3 is validated.

**(3) Path 3: “Academic burnout**→**Learning satisfaction**→**Learning engagement**→**Learning achievement”**

Referring once again to Table 4, the path from learning satisfaction to learning engagement is 0.3060, with a p-value of 0.0000, confirming a strong positive effect. This suggests that as students' learning satisfaction increases, their engagement with learning also intensifies, leading to better academic outcomes. With a 95% confidence interval of [-0.135, -0.028], the effect size for Path 3 is 0.075, and the interval excludes zero, affirming the existence of a significant mediation effect. These findings support the chain mediation role of both learning satisfaction and learning engagement. Therefore, Hypothesis H4 stands validated.

This study examined the chain mediation effects of academic burnout on learning satisfaction, learning engagement, and academic achievement. The regression analysis results are summarized in Table 5, which supports the hypothesized mediation model.

To ensure the robustness of the results, sensitivity analysis was conducted using three model configurations. As shown in Table 6, the relationships remained consistent across all configurations, with key path coefficients being statistically significant (*p* < 0.001) and 95% confidence intervals excluding zero, providing strong support for the internal validity of the chain mediation model.

**Table 6:** Sensitivity analysis.

**Experiment**	**AB → AA(*p*)**	**LS → LE(*p*)**	**LE → AA(*p*)**	**R^2^**
Experiment 1	-0.2338 (*p* < 0.001)	0.1952 (*p* < 0.001)	0.3272 (*p* < 0.001)	0.5103
Experiment 2	-0.2309 (*p* < 0.001)	0.1952 (*p* < 0.001)	0.3272 (*p* < 0.001)	0.5113
Experiment 3	-0.2309 (*p* < 0.001)	0.1952 (*p* < 0.001)	0.3272 (*p* < 0.001)	0.5103

Experiment 1 adds control variables such as gender and academic year; Experiment 2 modifies the order of the mediating variables; Experiment 3 serves as the baseline model.

#### 4.3.3 Moderating effects test

Relevant theories suggest that students' educational values may significantly influence their engagement with academic tasks and academic performance (Yu et al., 2024). These values provide a motivational framework that can amplify or weaken the effect of learning engagement on academic outcomes (Zhang et al., 2021). Given the positive correlation between educational values and learning outcomes, we hypothesize that they play a key role in moderating the impact of engagement on academic achievement. The experimental data were meticulously organized, and Models 1, 2, and 3 were constructed, as shown in Table 7, to explore the moderating role of educational values. The data presents the following striking findings:

**Table 7:** Moderating effects of educational values.

**Model**	**COEFF**	**SE**	***t***	***P***	**LLCI (95% CI)**	**ULCI (95% CI)**
Constant	4.007	0.177	22.692	0.000	3.660	4.355
1 (Learning engagement)	0.439	0.042	10.462	0.000	0.356	0.521
2 (Educational values)	0.448	0.052	8.580	0.000	0.346	0.551
3 (Interaction term)	0.111	0.046	2.412	0.016	0.020	0.201

SE stands for Standard Error, LLCI refers to the Lower Limit of the Confidence Interval, and ULCI refers to the Upper Limit of the Confidence Interval.

The effect of learning engagement on learning achievement stands at 0.439, with a 95% confidence interval of [0.356, 0.521], excluding zero. The *p*-value is 0.000 (*p* < 0.001), reinforcing the powerful link between learning engagement and academic performance. This indicates a significant positive relationship, where higher learning engagement is associated with improved academic achievement.

Educational values demonstrate a solid effect on learning achievement, with a coefficient of 0.448, a confidence interval of [0.346, 0.551], and a *p*-value of 0.000 (*p* < 0.001). This suggests a strong, positive relationship between educational values and student success.

The interaction term between educational values and learning engagement yields a coefficient of 0.439, with a 95% confidence interval of [0.020, 0.201], again excluding zero. The *p*-value of 0.016 (*p* < 0.001) reveals a significant moderating effect of educational values on learning engagement. Therefore, Hypothesis H4 is supported. That is, even with similar learning engagement, students' academic outcomes may differ due to educational values. Students with higher educational values tend to align their learning with personal goals, leading to better results. This indicates that educational values strengthen the link between engagement and achievement.

**Figure 3:**
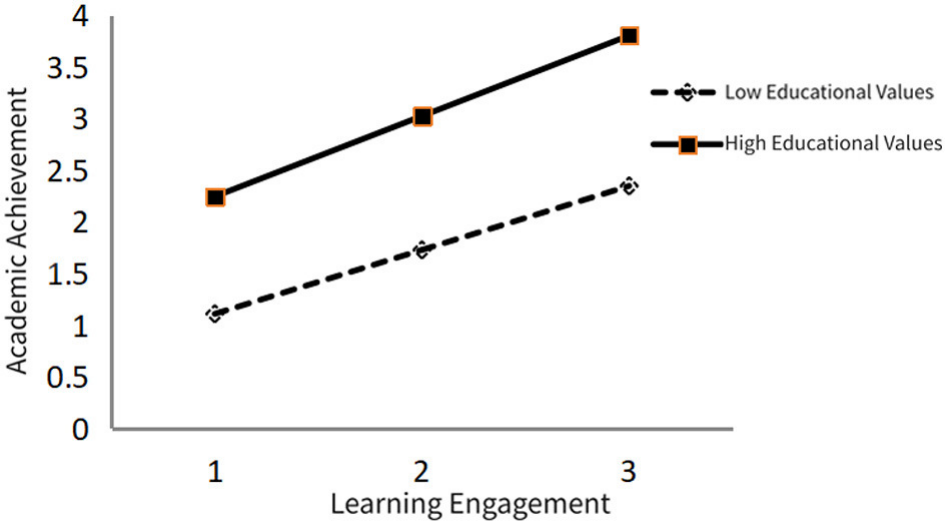
Effect of educational values on the relationship between learning engagement and academic achievement.

Further exploration of this moderating effect's mechanism led to the creation of a graphical representation, as depicted in Figure 3. The results illuminate a clear, significant moderating role played by educational values in the connection between learning engagement and academic success. Most notably, the slope for students with high educational values is considerably steeper than that for students with low educational values. This suggests that, under high educational values, students are more likely to appreciate the significance of learning engagement. Their efforts, thus, transform into higher academic achievement. Conversely, students with lower educational values are less likely to recognize the value of learning engagement, causing a weaker impact on their academic success. This reflects an underlying mechanism to some extent: students with stronger educational values are more likely to internalize the meaning of academic effort, viewing learning as a key pathway to achieving personal goals, thereby amplifying the impact of engagement on academic achievement.

#### 4.3.4 Moderated chain mediation effect

Expanding on the finding that educational values moderate the link between learning engagement and academic achievement, this study further investigates how educational values moderate the chain mediation effect (as detailed in Table 8). The results are compelling: the effect size for the chain mediation mechanism is 0.157, with a *p*-value of 0.014 (*p* < 0.01), indicating robust statistical significance. Moreover, the 95% confidence interval derived from the Bootstrap method does not include zero, firmly establishing the moderating effect.

**Table 8:** Effect of educational values in moderated mediation model.

**Path**	**Moderation level**	**COEFF**	**SE**	***p* value**	**LLCI**	**ULCI**
Academic burnout → Learning satisfaction → Learning engagement → Academic achievement	Low (-1SD)	-0.104	0.068	0.125	-0.236	0.028
Academic burnout → Learning satisfaction → Learning engagement → Academic achievement	Average	-0.157	0.064	0.014	-0.282	-0.032
Academic burnout → Learning satisfaction → Learning engagement → Academic achievement	High (+1SD)	-0.210	0.086	0.015	-0.378	-0.041

SD is the abbreviation for Standard Deviation.

In addition, the analysis reveals that at both high and low levels of educational values, the 95% confidence intervals exclude zero. This consistency further confirms the existence of a significant moderating role. These findings strongly validate the hypothesis that educational values play a critical role in shaping the chain mediation mechanism, thereby confirming the moderated chain mediation effect. As a result, Hypothesis H5 is supported without reservation.

## 5 Conclusion and future directions

### 5.1 Research conclusions

This study, rooted in the S-O-R theoretical framework, explored the mediating and chain mediating roles of learning satisfaction and learning engagement in the relationship between academic burnout and learning achievement, as well as the moderating effects of educational values. The conclusions drawn are as follows:

Academic burnout exerts a significant negative influence on learning achievement, revealing the detrimental effects of burnout on students' academic performance.

Both learning satisfaction and learning engagement serve as significant mediators between academic burnout and learning achievement. Furthermore, they collectively form a chain mediation pathway, wherein academic burnout reduces learning satisfaction, leading to diminished engagement and, ultimately, poorer academic outcomes.

Educational values act as a critical moderator, amplifying the positive impact of learning engagement on academic achievement. Students with stronger educational values benefit more from their engagement, which translates into higher academic performance and enhanced developmental outcomes.

The moderating effects of educational values extend to the chain mediation mechanism. Specifically, students with higher educational values exhibit a stronger chain mediation effect, which mitigates the negative impact of academic burnout and promotes superior academic achievement.

### 5.2 Theoretical contributions

First, this study introduces and constructs a comprehensive theoretical model based on the S-O-R framework, elucidating the pathways through which academic burnout affects learning achievement. By doing so, it enriches the existing body of research, which has predominantly focused on academic burnout as an outcome variable, and shifts the focus to academic burnout as a significant predictor. This shift is critical for understanding the proactive role burnout plays in shaping students' learning achievements and behaviors. On one hand, it highlights the detrimental impact of academic burnout on learning satisfaction and engagement, extending the theoretical understanding of burnout's negative effects. On the other hand, it delves into the mechanisms by which burnout depletes students' psychological and emotional resources, ultimately hindering their academic success. This dual focus not only deepens theoretical insights but also provides a practical foundation for addressing burnout in educational settings.

Second, the study uncovers the dynamic interplay between emotional and cognitive processes through the chain mediation of learning satisfaction and learning engagement. Unlike previous research, which often examined these factors in isolation, this study integrates them into a cohesive framework. Specifically, it validates the pathway: “Academic Burnout (S) → Learning Satisfaction (O, Emotional Cognition) → Learning Engagement (O or R, Behavioral Cognition) → Learning Achievement (R, Behavioral Outcome).” By bridging emotional and cognitive dimensions, the research reveals the intricate, systemic process through which burnout impacts academic outcomes, offering a richer, more nuanced perspective.

Finally, the study emphasizes the pivotal role of educational values in moderating the effects of academic burnout on learning achievement. Educational values not only enhance the relationship between learning engagement and achievement but also amplify the chain mediation pathway. For instance, students with high educational values experience stronger mediating effects of satisfaction and engagement, enabling them to counteract the negative effects of burnout more effectively. This finding broadens the scope of research on high academic achievement and underscores the importance of cultivating positive educational values. In the context of Chinese higher education, educational values' moderating role may be particularly strong, as academic achievement is highly valued, and students' educational values are deeply rooted in societal expectations and family influence. Future research could investigate how Chinese cultural factors, such as the importance placed on family support and academic success, contribute to the strength of educational values in moderating academic burnout.

### 5.3 Practical implications

Based on the above conclusions, several actionable recommendations are proposed:

**Improve students' learning satisfaction:** Learning satisfaction plays a key role in alleviating academic burnout and enhancing learning achievement. Universities should take a multi-faceted approach to improving students' learning experiences. Specific strategies include improving teaching quality, providing more personalized academic counseling, and fostering a supportive learning environment that promotes student well-being. Additionally, implementing stress-relief programs and creating platforms for student-peer interaction can significantly alleviate academic burnout. First, optimizing the allocation of educational resources and improving service quality, such as upgrading teaching facilities, diversifying course content, and enhancing administrative efficiency, can meet students' expectations for their learning environment. Second, teachers should focus on interaction and feedback during instruction, using methods like case studies, group discussions, and experiential learning to spark students' interest and increase engagement. Finally, initiatives like student feedback mechanisms, the refinement of teaching practices, and recognition of academic achievements can foster a stronger sense of belonging and encourage students to engage more deeply with their studies.

**Enhance learning engagement:** As a critical link between satisfaction and achievement, learning engagement is essential for translating educational values into actionable behavior. Schools should implement strategies to systematically boost students' focus and motivation. For instance, teachers can design clear learning objectives, provide phased incentives, and create an environment that sustains students' enthusiasm for academic tasks. Additionally, organizing academic and extracurricular activities–such as research projects, innovation challenges, internships, and field studies–can cultivate a spirit of inquiry and active participation. Furthermore, teaching time management and study techniques can empower students to adopt effective learning strategies, directly boosting their engagement and academic outcomes.

**Cultivate educational values:** Educational values significantly moderate the relationship between learning engagement and achievement, underscoring their critical role in driving academic success. Universities should foster these values through a variety of initiatives. Building a campus culture that emphasizes the societal and personal value of education, using lectures, forums, and themed activities, can help students internalize these ideals. Incorporating value education into daily instruction and campus life–via student organizations, community service, and collaborative projects–can further enhance students' appreciation for learning. In the context of Chinese higher education, educational values are deeply influenced by cultural norms, such as respect for teachers and family expectations for academic success. These values can be nurtured through initiatives like family education workshops and mentorship systems. Moreover, offering programs like personal development workshops and educational campaigns can help students connect their academic pursuits with broader social and personal goals, ultimately embedding educational values into their mindset.

### 5.4 Limitations and future directions

Despite its contributions, this study has certain limitations:

**Mediating mechanisms:** This study only considers the mediating roles of learning satisfaction and learning engagement. Other potential mediators, such as academic self-efficacy, time management skills, psychological resilience, and social support, were not included. Future research could explore these additional factors to provide a more comprehensive understanding of the mechanisms linking academic burnout to learning achievement.

**Methodological considerations:** Although this study provides valuable insights into the relationships between academic burnout, learning engagement, and achievement, its cross-sectional design limits the ability to make causal inferences. Future research could consider employing longitudinal data or experimental designs, which would enhance the ability to draw causal conclusions about the relationships among these variables.

**Moderating variables:** While the moderating effect of educational values was examined, other contextual variables–such as discipline type or family support–were not addressed. Future studies could investigate how these factors influence the relationship between academic burnout and learning achievement, revealing both generalizable and context-specific mechanisms.

**Sample scope:** The sample in this study was limited to university students from a specific region in China. Variations in educational environments, academic pressures, and cultural backgrounds across regions may limit the generalizability of the findings. Expanding the sample to include students from diverse regions and cultures would enhance the applicability and robustness of the conclusions.

## Data Availability

The datasets used and/or analyzed during the current study are not publicly available because they contain sensitive personal and academic information of student participants, and the informed consent obtained restricts public sharing. Requests to access the datasets should be directed to the corresponding author, Ziqian Lin (linziqian@gxjcxy.edu.cn).
